# Data Integration Reveals the Potential Biomarkers of Circulating MicroRNAs in Osteoarthritis

**DOI:** 10.3390/diagnostics11030412

**Published:** 2021-02-28

**Authors:** Thuan Duc Lao, Thuy Ai Huyen Le

**Affiliations:** Faculty of Biotechnology, Ho Chi Minh City Open University, Ho Chi Minh City 700000, Vietnam; thuan.ld@ou.edu.vn

**Keywords:** osteoarthritis, circulating miRNAs, miRNAs-based diagnosis, miRNAs-based therapeutic

## Abstract

The abnormal expression of circulating miRNAs (c-miRNAs) has become an emerging field in the development of miRNAs-based diagnostic and therapeutic tools for human diseases, including osteoarthritis (OA). OA is the most common form of arthritis leading to disability and a major socioeconomic burden. The abnormal expression of miRNAs plays important roles in the pathogenesis of OA. Unraveling the role of miRNAs in the pathogenesis of OA will throw light on the potential for the development of miRNAs-based diagnostic and therapeutic tools for OA. This article reviews and highlights recent advances in the study of miRNAs in OA, with specific demonstration of the functions of miRNA, especially c-miRNA, in OA pathogenesis as well as its potential implication in the treatment of OA. Based on a systematic literature search using online databases, we figured out the following main points: (1) the integrative systematic review of c-mRNAs and its target genes related to OA pathogenesis; (2) the potential use of c-miRNAs for OA diagnosis purposes as potential biomarkers; and (3) for therapeutic purposes, and we also highlight certain remedies that regulate microRNA expression based on its target genes.

## 1. Introduction

Osteoarthritis (OA) is the most common degenerative joint disease and the leading cause of pain and disability, affecting more than 25% of the adult population [1,2,3]. Worldwide, 10% of men and 18% of women aged 60 years and above are diagnosed with OA [4]. In addition, OA is considered a tremendous individual and socioeconomic burden as a result of loss of productivity and increased medical costs, leading to reduced quality of life [5,6]. Both exogenous and endogenous factors have been identified as etiological factors of OA [7,8] (Table 1).

The primary clinical symptoms are joint pain (history of joint pain), stiffness, locomotor restriction, muscle weakness, and poor balance, which guide the diagnosis. To date, radiography remains the gold standard for diagnosing OA [9]. However, the gold standard diagnosis has limitations when assessing and monitoring the early stage of OA [1,9]. Other than joint surgery-based treatment, there is no cure for OA, and most treatments focus on the relief of symptoms such as pain. However, side effects from pain medications, such as dizziness, which contributes to falls, have been reported during therapy. Therefore, improved identification of potential biomarkers that promote OA progression as well as therapeutic strategies are not only essential for the development of new diagnosis strategies, but also for promising treatment outcomes.

Research programs related to the development of early diagnosis tools and monitoring the progression of OA based on biomarkers have gained considerable attention in recent years. In a recent study, 4191 OA patients (41.88% male and 58.12% female) were recruited to identify potentially clinically useful biomarkers of OA [30]. The data showed that of the biomarkers, including serum C1, C2, serum C2C, serum CPII, serum PIIANP, serum Coll21 NO2, serum CS846, serum MMP3, serum CTXI, serum COMP, serum HA, serum NTXI, urine CTXII, urine C1, C2, urine C2C, urine NTXI, urine CTXI alpha, urine CTXI beta, urine Coll21 NO2, and urine creatinine, only four, namely, serum Coll21 NO2, serum CS846, serum COMP, and urine CTXII, were reported to be consistently associated with OA, thus providing promising potential biomarkers of OA [30]. Among these four potential biomarkers, the urine CTXII was identified as the biomarker with the strongest and most significant association with features of OA [30]. Based on further investigation, these four biomarkers may be clinically useful surrogates that can be used for the development of biomarkers of OA in the near future. Molecules that exist in joint fluid have also attracted interest in the investigation of biomarkers for OA. For example, 159 OA patients, a total of 138 patients returned for follow-up 3 years later to assess progression of knee OA, and the study found that the *NLRP3* gene was activated by uric acid. When activated, this leads to the production of IL-18 and IL-1β. Synovial fluid uric acid and IL-18 were strongly and positively associated with OA severity, and synovial fluid IL-1β was associated with OA severity. Additionally, synovial fluid IL-18 was identified to be associated with a 3-year change in OA severity. These results strongly support the potential involvement of the innate immune system in OA pathophysiology. Therefore, synovial fluid uric acid is a marker of knee OA severity [31]. Even though there are currently no biomarkers approved by the FDA for the diagnosis and monitoring of OA due to limited specificity and sensitivity, such advanced research in the OA biomarker field is still progressing steadily [30,32]. The majority of nucleic acid-based molecules, such as circulating miRNAs (c-miRNAs), exist in different biological fluid types, such as blood, serum, plasma, saliva, tears, urine, milk, follicular fluid, semen, etc., and they have shown great potential for application of circulating biomarkers for early diagnosis since they can be secreted into circulation and are stable. Several studies have indicated that the selective expression of c-miRNAs plays a key role in the progression of OA. In this review, we focus on the compilation of studies regarding the expression of c-miRNAs in OA. These abnormal expressions of c-miRNAs could be used as potential biomarkers to improve current diagnostic practices performed in the clinic to detect and monitor OA.

## 2. Brief Introduction to miRNAs and c-miRNAs

Epigenetic modification, including DNA methylation, histone modification and microRNAs (miRNAs), refers to the heritable changes in gene expression without any changes in DNA sequence [33]. Since the discovery of miRNA, *lin-4*, in 1913 by Ambros and Ruvkun in *Caenorhabditis elegans*, miRNAs have attracted attention as potential biomarkers of several human diseases, including OA. Mature miRNAs are single-strand non-coding RNA with lengths of 19–25 nucleotides [1,34]. The biogenesis of miRNA is classified into two categories: (1) the canonical pathway, and (2) the non-canonical pathway [35]. miRNAs function via base-pairing with their target gene to modulate the target gene expression, which plays various roles in diverse processes of living, including cell proliferation, differentiation, metabolism, stress response and apoptosis [36]. Compared with mRNA, an unexpected characteristic of miRNA is the stability of the short sequences that are detected in the cellular microenvironment [37]. Recently, a handful of miRNAs, known as c-miRNAs or extracellular miRNAs, were detected in the outside environment of the cell (extracellular environment), including blood, serum or plasma [1,37]. Additionally, not only were they detected in blood, serum or plasma, c-miRNAs were also detected in different biological fluid types, such as saliva, tears, urine, milk, follicular fluid, semen, etc. [1,37,38]. Accumulating evidence has shown that c-miRNAs are resistant to degradation by ribonuclease activities in plasma or any other biological fluids, suggesting that they are protected in a specific manner, that is, packing in small membrane vesicles, such as exosomes, microvesicles, apoptotic bodies, RNA-binding proteins (AGO-2), nucleophosmin I, and lipoprotein complexes (such as high-density lipoprotein (HDL)) [35,37,39,40]. In addition to the property of resistance to RNase activity, one of the key molecular characteristics of c-miRNAs is high stability in extreme physiological conditions, such as high or low pH, and different variations, such as boiling and multiple freeze-thaw cycles [39,41]. Aberrant expression of miRNAs, as well as c-miRNAs, has been linked to many human diseases, such as inflammatory diseases [39,42], cancers [43,44,45], immune-related diseases [46], neurodegenerative diseases [47], etc., including OA. However, data concerning their detection could be controversial since miRNAs, as well as c-miRNAs, are not specific to certain diseases; however, they may add to the procedure of diagnosis and therapeutic approaches in combination with other markers, such as their downstream target genes, to build a specific signature for a certain disease. It is noted that more than 50% of all genes in the human genome are reported to be the target genes of miRNAs/c-miRNAs, thus resulting in the regulation of manifold metabolic and regulatory pathways such that the integrative network between miRNA/c-miRNAs and its target genes becomes more and more possible [48,49]. Hence, the abnormal expression profiles of miRNAs could be applied in the management of certain human diseases.

Remarkable studies have been published about c-miRNAs’ dysregulated expression correlated with the pathogenesis of OA, such as osteoarthritic cartilage apoptosis, autophagy, chondrogenesis and cartilage homeostasis, matrix metalloproteinases and aggrecanases, inflammation, etc. In this review, we summarize the studies that have revealed a link between c-miRNA and OA by shedding light on c-miRNAs and their possible functions, in addition to commenting on their potential as noninvasive biomarkers of OA.

## 3. c-miRNA Signature as Potential Biomarker for OA Diagnosis

In order to understand the extent to which c-miRNAs are involved in the processes of pathogenesis, we analyzed the results of many recent studies focused on how c-miRNAs regulate those events that are germane to OA pathophysiology. A number of c-miRNAs have been found to be involved in the pathogenesis of OA (Table 2).

Further, many c-miRNAs have been found to be overexpressed in OA, including miR-146a, miR-155, miR-181, miR-223, miR-16, miR-20, miR-30, miR-126, miR-184, miR-186, miR-195, miR-345, miR-885, miR-122, miR-23a-3p, miR-24-3p, miR-27b-3p, miR-29c-3p, miR-34a-5p, miR-92-3p, and miR-486-5p (Table 2). One of the most studied overexpressed c-miRNAs is miR-146a [50,51,52,53].

**Figure 1:**
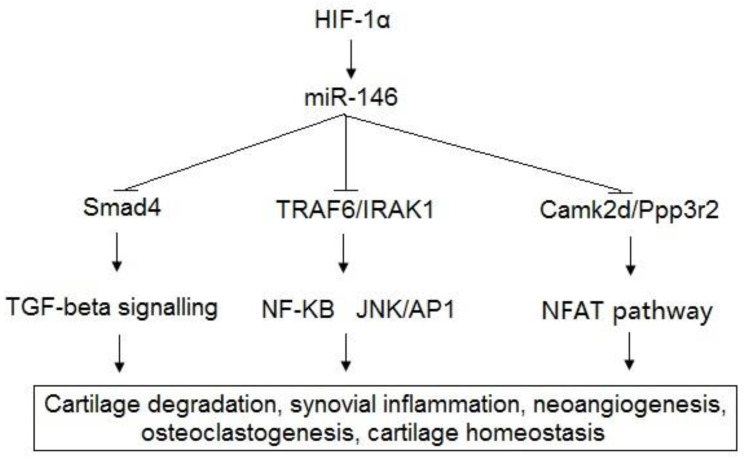
Schematic representation of miR-146 and its target genes related to pathogenesis of OA.

As described, miRNAs are not specific to certain diseases; miR-146a has also been reported to be linked to many human diseases, such as Type II diabetes [60], systemic lupus erythematosus [61,62], cancers [63,64], etc. The complex target networks pose a significant challenge to establishing a specific signature for a certain disease. Higher expression of miR-146a and its integrative network have been reported to be correlated with the pathogenesis of OA. miR-146a-5p was significantly overexpressed in OA patients in samples of cartilage (*p* = 0.006) and serum (*p* = 0.002), which were analyzed by qPCR for 28 OA patients compared with those of two healthy controls [52]. The expression levels of miR-146a-5p in the serum were positively correlated with those of the cartilage (Pearson correlation coefficient, R = 0.32; *p* = 0.002) [52]. With a next-generation sequencing approach and qPCR, Rousseau et al. concluded that serum miR-146a-5p was significantly overexpressed in a group of OA patients compared with the controls [53]. Therefore, they suggested that miR-146a-5p serum level could serve as a non-invasive clinical utility biomarker for OA management. It has been determined that the function of miR-146a is linked to cartilage degradation, synovial inflammation, neoangiogenesis, and osteoclastogenesis [52]. To support the understanding of the OA mechanism, the target genes of miR-146a have been determined for OA pathophysiology. The target genes of miR-146a, including Bcl-2, TRAF6, IRAK1, VEGF, Smad4, and TGF-β, have been identified in previous studies [50,51,52,53,65,66,67,68,69]. Autophagy was reported to be induced by hypoxia via miR-146a, and Bcl-2 [65]. Moreover, miR-146a promotes chondrocyte autophagy by inhibiting Bcl-2, an autophagy inhibitor [65]. The inhibition of Bcl-2 by hypoxia-induced miR-146a has been found to promote chondrocyte autophagy through the pathway of TRAF6/IRAK1 [66]. Furthermore, a study reported that miR-146a was increased by mechanical pressure injury, and its upregulation induced the apoptosis of human chondrocytes via inhibition of Smad4 in the cartilage by harboring a miR-146a binding sequence in the 3′-UTR of its mRNA [67]. The function of Smad4 in the chondrocytes is to serve as the mediator of the upregulation of miR-146a-induced VEGF subjected to mechanical pressure injury. The results of the study indicated that miR-146a plays a key role in the apoptosis of human chondrocyte, thereby contributing to the pathogenesis of OA by increasing the levels of VEGF and damaging the transforming growth factor (TGF)-β signaling pathway through the targeted inhibition of Smad4 in the cartilage [67]. The rupture of cartilage homeostasis results in the induction of phenotypic modifications of chondrocytes, leading to cartilage damage and pathogenesis of OA [68]. Cartilage homeostasis has been reported to be regulated by miR-146a through the suppression of cartilage matrix-associated gene expression, including Camk2d and Ppp3r2 [69]. miR-146a inhibitor-based treatment of surgically-induced OA mice was shown to significantly alleviate the destruction of articular cartilage via targeting of Camk2d and Ppp3r2 [69]. (Figure 1).

Other miRNAs were also reported to be significantly correlated with the pathogenesis of OA. The levels of 380 plasma miRNAs in patients with OA and healthy controls were compared. The results indicated there were 12 overexpressed detectable miRNAs, including miR-16, miR-20b, miR-29c, miR-30b, miR-93, miR-126, miR-146a, miR-184, miR-186, miR-195, miR-345, and miR-885-5p, that were altered in the OA and could be released into the plasma [54]. Moreover, in their report, they indicated that, among them, the expression of 8 miRNAs, including miR-29c, miR-93, miR-126, miR-184, miR-186, miR-195, miR-345 and miR-885, was confirmed as the high expression of c-miRNAs, presenting a three- to four-fold change in patients with OA based on qPCR analysis [54]. The putative target mRNA genes of these miRNAs, such as FGFRR1, HDAC4, FGF2, VEGFA, IGFIR, ADAMTS5, TIMP2, and WISP1, were differentially expressed in OA. All the targets are important in OA because they take part in diverse signaling pathways including chondrocyte maintenance, osteocyte modulation, and chondrocyte inflammation [54]. Furthermore, six miRNAs, including miR-23a-3p, miR-24-3p, miR-27b-3p, miR-29c-3p, miR-34a-5p and miR-186- 5p, were significantly upregulated in late-stage OA synovial fluid compared with early-stage OA synovial fluid [55]. Among them, miR-29c and miR-186 have been reported to be overexpressed in the plasma of early-stage OA synovial fluid compared with healthy controls [55]. Overall, the identification of such patterns of c-miRNAs in body fluids suggests that the significant difference between late-stage and early-stage OA may help in examining the outcomes of disease to develop the diagnosis as well as the therapeutics of OA in the future. Mounting evidence suggests that miR-186 over-expression is involved in the pathogenesis of OA; however, controversies regarding miR-186 expression in OA still exist. In other study, which aimed to study the mechanism of miR-186 in OA, the expression levels of miR-186 were measured in the cell in vivo using q-PCR. Their results indicated that miR-186 was remarkably decreased in comparison with the control group. Interestingly, miR-186 was shown to increase chondrocyte survival, facilitate cell cycle entry in OA chondrocytes, and inhibit the PI3K–AKT pathway via SPP1-inhibited chondrocyte apoptosis in mice with OA. Notwithstanding these controversies, these results have opened up an approach to early diagnosis and prognosis of OA [70].

The downregulation of miRNAs, including miR-132, miR-25, miR-28, miR-140, miR-191, miR-342, miR-454, miR-let-7b, miR-let-7a, miR-27a, miR-329, miR-708, miR-934, miR-877, miR-1180, miR-320b, and miR-663a, has been identified in the progression of OA (Table 2). Another miRNA that has been studied in OA is miR-140. Its expression was decreased in osteoarthritic cartilage compared with healthy cartilage [1,56,71]. In other study, serum analysis was performed on 12 primary OA patients, compared with 12 healthy individuals, and the results indicated that miR-140 expression levels were consistently downregulated in articular cartilage of OA patients [56]. In their report, InsR and IGFR were identified as the common targets of miR-140, which are both involved in the regulation of metabolic processes contributing to the pathophysiology of OA [56]. Additionally, ADAMTS5, MMP-13, IGFBP5, and RALA, which play important roles in mediating the degradation of cartilage matrix, modulating the availability of IGF-1 in joint and regulating cartilage matrix development, have been identified as targets of miR-140 [71,72,73]. Transgenic mice miR-140^−/−^ were designed that showed age-related OA-like changes characterized by proteoglycan loss and fibrillation of articular cartilage [71]. Furthermore, the transgenic mice with overexpression of miR-140 were reported to be resistant to antigen-induced arthritis via the regulation of ADAMTS5, a major cartilage matrix-degrading protease in OA. Their findings demonstrated that miR-140 was required for the development of skeletal as well as cartilage homeostasis, and protected against OA-like pathophysiology via the regulation of ADAMTS5 [71].

Furthermore, several targets of miRNAs in the progression of OA, which play various roles in apoptosis, autophagy, chondrogenesis and cartilage homeostasis, matrix metalloproteinases and aggrecanases, inflammation, etc., have also been identified in numerous studies [1,34,53,74,75]. Our literature search identified a wide range of target genes of miRNAs that have been indicated to be associated with OA progression, as summarized in Table 3.

The discussion above shows that many previous studies have highlighted the possible application of c-miRNAs as well as miRNAs as novel biomarkers of OA. Hence, studying the dysregulation of miRNA and its integrative network provides the hope that, in the near future, miRNAs could serve as biomarkers of OA.

## 4. Therapeutic Potential of c-miRNAs in OA

Currently, miRNA-based therapeutics have been developed for the treatment of a variety of human diseases. Three miRNA-based therapeutic approaches have been suggested: (1) miRNA sponge-expression vector, (2) small molecule inhibitor, and (3) antisense oligonucleotides (ASO) [97]. Even though miRNA-based therapeutic drugs have not been commercially available, many miRNA-based drugs are in the process of development by numerous pharmaceutical and biotech companies. Some of these include RG-101 (targeted miRNA: miR-122; Phase 2; Company: Regulus therapeutics, regulusrx.com) for HCV therapy; MRG-106 (targeted miRNA: miR-155; Phase 1 and Phase 2; Company: MiRagen Therapeutics, miragentherapeutics.com) for lymphoma and leukemia therapy; and Mesomir (targeted miRNA: miR-16; Phase 2; Company: ENGeneIC; Phase 2, engeneic.com) for mesothelioma therapy.

miRNAs, as well as c-miRNAs, have been proposed as potential therapeutic candidates to ameliorate human diseases, including OA, through addressing the signature profile of dysregulation of miRNAs. If an upregulated miRNA contributes to the pathogenesis of OA, the inhibition therapies of that miRNA could be used to block it [98]. However, clinical success in the regulation of these molecules has been inadequate so far. An example of miRNA-target therapy of OA is the inhibition of miR-34a by the LNA-ASO method in mice [99]. In a study, the expression level of miR-34a-5p was reported to be significantly increased in human plasma, cartilage and synovium of late-stage OA patients and in the cartilage and synovium of mice subjected to surgical destabilization of the medial meniscus (DMM). Using an in vivo-grade, five-microgram of miR-34a-5p LNA-ASO was injected into the knee joints of mice at two, four and six weeks post DMM surgery. The results indicate that miR-34a-5p LNA-ASO prevented the degeneration of articular cartilage by the reduction of proteoglycan loss, chondrocyte loss, cartilage fibrillation, as well as the reduction of CASPASE-3, PARP p85 and MMP-13 expression, compared with control-injected mice [99]. Thus, the pre-clinical data from the study confirmed that miR-34a-5p LNA-ASO-based therapy has the potential to protect against DMM-induced cartilage damage [99]. Endisha et al. demonstrated that the injection of miR-34a-5p ASO imparts cartilage-protective effects in a mice model [99]. In a previous study reported that the injection of anti-miR-34a encoded lentiviral vector ameliorated the progression of OA by the regulation of the SIRT1/p53 signaling pathway [100]. Furthermore, it is possible that targeting the gene network of miRNAs can lead to positive effects in the treatment of OA. The axis of miR-122/SIRT1 regulated the chondrocyte extracellular matrix degradation, thus, providing new insights into the treatment of OA [101]. A study focused on the use of resveratrol (trans-3-4-5-trihydroxystilbene), which is targeted at SIRT1, to prevent the progression of OA [102]. In the study, the administration of resveratrol significantly induced the activation of SIRT1, thereby silencing the expression of HIF-2 α in mouse OA cartilage, preventing the progression of OA [102]. Therefore, it emphasizes the mechanisms underlying the activities of alternative remedies and their role in regulating miRNAs linked to OA progression, leading to the development of the miRNA-based therapy for OA.

## 5. Conclusions

In summary, as described in the article, multiple studies have demonstrated that the abnormal expression of miRNAs potentially plays an important role in the pathogenesis of OA by regulating many diverse processes, including cartilage degradation, synovial inflammation, neoangiogenesis, osteoclastogenesis, and cartilage homeostasis. Also, specific dysregulated miRNAs, c-miRNAs, could be released into body fluids, such as plasma, peripheral blood and synovial fluid, and these c-miRNAs are potential biomarkers of OA. On the other hand, the development of miRNAs-based diagnostic and therapeutic tools for human diseases, including OA, is definitely a long process. It is noted that the increasing evidence of miRNAs’ involvement in OA signals is the beginning of this long process. Therefore, it is necessary to perform validation analysis for the development of miRNAs-based therapeutics for OA.

## Figures and Tables

**Table 1:** Endogenous and exogenous risk factors of osteoarthritis (OA).

**Endogenous Factors**	**Comments**
Age	Increasing incidence rate in younger adults. Over half of people diagnosed are under 65 years of age [10,11].
Gender	OA is more common in women than men, particularly after menopausal age [12,13].
Ethnicity	OA is more common in African-Americans compared to other ethnic groups [14].
Genetics, Epigenetics	Genetic loci, multigene interaction, family heredity, methylation, histone modification, microRNA [7,8,15,16,17,18].
**Exogenous Factors**	**Comments**
Knee events	Injury, joint surgery, knee pain, trauma or repeated loading [7,8,19].
Obesity	BMI > 30 kg/m^2^ were 6.8 times more likely to develop knee OA than normal-weight controls [20].
Lifestyle factors	Tobacco, alcohol assumption [21,22].
Nutrition	Metabolic disease, such as lipid and cholesterol profiles, adequate vitamin levels, etc., essentially contribute to OA [23,24].
Occupation, sports	The link between occupational activities, including kneeling, squatting, lifting, climbing, heavy stand working, heavy physical load work or combinations thereof, and OA has been reported [25,26,27]. Thus, many occupations have been reported to be linked to OA, such as floor layers, miners, dockers, carpenters, firefighting, mining asphalt, plumbers, bricklayers, sports at elite levels, etc. [28,29].

**Table 2:** The dysregulation of circulating miRNAs (c-miRNAs) in the pathogenesis of OA.

References	c-miRNAs	Sources
[1]	miR-122↑, miR-25↓, miR-28-3p↓, miR-93↓, miR-140↓, miR-191↓, miR-342-3p↓, miR-146b↓, miR-454↓, miR-885-5p↑, miR-let-7b↓, miR-let-7e↓	Serum
[50]	miR-146a↑, miR-155↑	Peripheral blood
[51]	miR-132↓, miR-146a↑, miR-155↑, miR-181↑, miR-223↑	Serum
[52]	miR-146a-5p↑	Serum
[53]	miR-146a-5p↑, miR-186-5p↑	Serum
[54]	miR-16↑, miR-20b↑, miR-19c↑, miR-30b↑, miR-93↑, miR-126↑, miR-184↑, miR-186↑, miR-195↑, miR-345↑, miR-885-5p↑	Plasma
[55] *	miR-23a-3p↑, miR-24-3p↑, miR-27a-5p↓, miR-27b-3p↑, miR-29c-3p↑, miR-34a-5p↑, miR-329↓, miR-655↓, miR-708-3p↓, miR-934↓ and miR-186-5p↑ miR-27a-3p, miR-101-5p, miR-378-5p only detected in the late stage of OA.	Synovium
[56]	miR-140-3p↓, miR-33b-3p↓, miR-671-3p↓	Serum
[57]	miR-132↓	Synovium, plasma
[58]	miR-122-5p↑, miR-92a-3p↑, miR-19b-3p↑, miR-486-5p↑, miR-877-5p↓, miR-1180-3p↓, miR-320b↓, miR-663a↓	Blood
[59]	miR-120↑	Synovium

Note: ↑ Upregulated, ↓ Downregulated; * c-miRNAs’ expressions were compared in the late stage of OA vs early stage of OA.

**Table 3:** Experimentally verified target genes of c-miRNAs involving OA.

References	c-miRNAs	Target Genes	Function
[50,51,52,53,65,66,67,68,69]	miR-146a	Bcl-2, TRAF6, IRAK1, VEGF, Smad4, TGF-β, Camk2d, Ppp3r2	Cartilage degradation, synovial inflammation, neoangiogenesis, osteoclastogenesis, cartilage homeostasis.
[37,41,76]	miR-155	ULK1, MAP1LC3, ATG14, SHIP1	Autophagy, inflammation
[77]	miR-181	PTEN	Apoptosis
[78]	miR-16	Smad3	Chondrocyte growth, differentiation
[79,80]	miR-30b	ERG, BECN1	Chondrocyte differentiation, autophagy
[81]	miR-126	Bcl-2	Inflammation
[70]	miR-186	SPP1	Chondrocyte apoptosis
[64,82]	miR-195	HIF-1α, REGγ	Chondrocyte apoptosis, inflammation
[83,84]	miR-23a	Smad3, RUNX2	Chondrocyte growth, cartilage homeostasis
[85]	miR-24	p16^INK4α^	Reduces production of the two matrix remodeling enzymes
[86,87]	miR-27b	MMP-13, CBFB	Matrix degradation, chondrocyte differentiation
[88,89]	miR-34a	Col2α1, iNOS, TGIF2	Chondrocyte apoptosis
[90]	miR-92a	HADC2	Cartilage development and homeostasis
[91,92]	miR-19b	EZH2, LncRNA H19	Chondrocyte apoptosis, ECM degradation
[93]	miR-486	Smad2	Chondrocyte growth
[56,71,72,73]	miR-140	InsR, IGFR, ADAMTS5, MMP-13, IGFBP5, RALA	Metabolic processes, cartilage homeostasis and chondrogenesis.
[94,95]	miR-25	COX2	Inflammation
[96]	miR-107	TRAF3	Chondrocyte apoptosis, autophagy

## Data Availability

Not applicable.

## Abbreviations

ADAMTS5	A disintegrin-like and metalloproteinase with thrombospondin-1 motifs 5
AGO	Argonaute protein
AKT	Serine/threonine-specific protein kinase
ATG14	Autophagy related 14
Bcl-2	B-cell lymphoma 2
BECN1	Beclin 1
BMI	Body mass index
C1, C2, C2C	Types I and II collagen marker degradation markers
Camk2d	Calcium/calmodulin-dependent protein kinase type II delta
CASPASE-3	Cysteine-dependent aspartate-directed proteases 3
CBFB	Core-binding factor subunit beta
c-miRNA	Circulating microRNA
Col2α1	The type II collagen gene
Coll2-1 NO2	The nitrated form of α-helical region of type II collagen
COMP	Oligomeric matrix protein
COX2	Prostaglandin-endoperoxide synthase 2
CPII	C-propeptide of type II collagen
CS846	Chondroitin sulphate 846
CTXI	C-terminal cross-linked telopeptide of type I collagen
CTXI alpha and CTXI beta	Alpha and beta isomerised versions of the CTXI
CTXII	C-terminal crosslinked telopeptide of type II collagen
DMM	Medical meniscus
ERG	Erythroblastosis virus E26 oncogene homolog-related gene
EZH2	Enhancer of zeste homolog 2
FGF2	Fibroblast growth factor 2
FGFR	Fibroblast growth factor receptor
HA	Hyaluronic acid
HADC2	Histone deacetylase 2
HDAC4	Histone deacetylase 4
HDL	High-density lipoprotein
HIF-1α	Hypoxia-inducible factor 1-alpha
HIF-2α	Hypoxia-inducible factor 2-alpha
IGFBP5	Insulin-like growth factor-binding protein 5
IGFIR	Insulin-like growth factor 1 receptor
IGFR	Insulin-like growth factor 1 receptor
IL	Interleukine
iNOS	Inducible nitric oxide synthase
InsR	Insulin receptor
IRAK1	Interleukin-1 receptor-associated kinase 1
LNA-ASO	Locked nucleic acid antisense oligonucleotide
LncRNA H19	Long non-coding RNA H19
MAP1LC3	Microtubule-associated proteins 1A/1B light chain 3B
miRNA	microRNA
MMP-13	Matrix metalloproteinase 13
MMP3	Matrix metalloproteinase-3
NLRP3	The Nacht, leucine-rich repeat and pyrin domain containing protein 3
NTXI	The cross-linked N-telopeptide of type I collagen
OA	osteoarthritis
p16INK4α	Cyclin dependent kinase inhibitor 2A
PARP p85	Poly (ADP-ribose) polymerase p85
PI3K	Phosphoinositide 3-kinases
PIIANP	N-propeptide of collagen IIA
Ppp3r2	Protein phosphatase 3 regulatory subunit B
PTEN	Phosphatase and tensin homolog
qPCR	Quantitative real-time PCR
RALA	Ras-related protein Ral-A
REGγ	Langerhans regenerating protein γ
RUNX2	Runt-related transcription factor 2
SHIP1	Src homology 2 domain containing inositol polyphosphate 5-phosphatase 1
SIRT1	Sirtuin 1
Smad2	Mothers against decapentaplegic homolog 2
Smad3	Mothers against decapentaplegic homolog 3
Smad4	SMAD family member 4
SPP1	Secreted phosphoprotein 1
TGF	Transforming growth factor
TGIF2	TGFB-induced factor homeobox 2
TIMP2	Tissue inhibitor of metalloproteinases 2
TRAF3	TNF receptor-associated factor 3
TRAF6	TNF receptor associated factor 6
ULK1	Unc-51-like autophagy activating kinase 1
VEGF	Vascular endothelial growth factor
VEGFA	Vascular endothelial growth factor A
WISP1	Wnt1-inducible signaling pathway protein 1
